# Characteristics Associated with COVID-19 Breakthrough Infections after Booster Vaccinations in Healthcare Workers: Insights from the TüSeRe:exact Study

**DOI:** 10.3390/jcm13061571

**Published:** 2024-03-09

**Authors:** Günalp Uzun, Alan Bareiß, Matthias Becker, Karina Althaus, Alex Dulovic, Daniel Junker, Katja Schenke-Layland, Peter Martus, Oliver Borst, Nicole Schneiderhan-Marra, Tamam Bakchoul

**Affiliations:** 1 Centre for Clinical Transfusion Medicine, 72076 Tübingen, Germanykarina.althaus@med.uni-tuebingen.de (K.A.); 2 Institute for Clinical and Experimental Transfusion Medicine, Medical Faculty, University Hospital Tübingen, 72076 Tübingen, Germany; 3 NMI Natural and Medical Sciences Institute at the University Tübingen, 72770 Reutlingen, Germany; 4 Institute of Biomedical Engineering, Department for Medical Technologies & Regenerative Medicine, Eberhard Karls University Tübingen, 72076 Tübingen, Germany; 5 Institute for Medical Biometrics and Clinical Epidemiology, Medical Faculty, University Hospital Tübingen, 72076 Tübingen, Germany; peter.martus@med.uni-tuebingen.de; 6 DFG Heisenberg Group Thrombocardiology, Eberhard Karl University Tübingen, 72076 Tübingen, Germany; 7 Department of Cardiology and Angiology, University Hospital Tübingen, 72076 Tübingen, Germany

**Keywords:** COVID-19, breakthrough infection, health personal, cardiovascular disease

## Abstract

**Background:** The prevalence of COVID-19 breakthrough infections in healthcare workers (HCWs) remains an issue of concern. This study examines the different characteristics associated with breakthrough infections in HCWs. **Methods:** From the total participants in the TüSeRe:exact study (n = 1046), we specifically included study participants who had received three vaccinations and were not infected prior to the third vaccination. Participants were invited to complete an online questionnaire, which included inquiries about any breakthrough infections they might have experienced. Univariate Cox regression analysis was used to investigate the association between participant characteristics and breakthrough infections. **Results:** Among 629 HCWs (497 female and 132 male), 241 (38%) experienced breakthrough infections during the follow-up period. The frequency of breakthrough infections was 39.2% (195/497) among female participants and 34.8% (46/132) among male participants (*p* = 0.357). The Cox regression model adjusted for age and sex showed that participants with cardiovascular disease (hazard ratio (95%CI) = 0.621 (0.392–0.985); *p* = 0.043) and those taking antihypertensives (hazard ratio (95%CI) = 0.551 (0.331–0.915); *p* = 0.021) had a significantly lower hazard ratio for breakthrough infections. The use of analgesics after the first vaccine (hazard ratio (95%CI) = 1.343 (1.025–1.759); *p* = 0.032) was associated with an increased risk of breakthrough infections. **Conclusions:** These findings can inform targeted preventive measures and risk management strategies to protect frontline workers and maintain a resilient healthcare system during the ongoing pandemic.

## 1. Introduction

As of the 15 November 2023, the global administration of over 13.5 billion vaccine doses against SARS-CoV-2 resulted in the vaccination coverage of 66% globally [1]. Vaccination leads to the development of antibodies against SARS-CoV-2, which can be detected continuously [2]. Although vaccine effectiveness against severe disease remains high six months after full vaccination, protection against symptomatic disease declines by approximately 20–30% over the same period [3]. Real-world data from Israel, as reported by Bar-On et al., also highlighted the efficacy of a third dose of an mRNA vaccine in significantly reducing the risk of infection and severe illness caused by the Delta variant [4]. While booster doses provide significant protection against severe SARS-CoV-2 infection [5], a persistent 10% severe infection rate remains among certain high-risk groups following breakthrough infections [6].

Healthcare workers are particularly at risk due to their frequent and close contact with infected patients, resulting in their high representation among COVID-19 cases prior to the availability of vaccines [7]. Breakthrough infections, referring to cases where vaccinated individuals become infected with SARS-CoV-2, were found to be significantly higher in nurses compared to other professional groups [8]. Due to waning immunity and the emergence of new variants, it is necessary to implement measures such as continuous screening and testing in healthcare workers.

Several studies have highlighted the association between comorbidities, medications, and breakthrough infections in COVID-19. Smits et al. [9] reported that patients with multiple comorbidities had a higher risk of COVID-19 breakthrough infections than those without these conditions. In addition, Schiff et al. demonstrated CD20i and glucocorticoid monotherapy as risk factors for COVID-19 breakthrough infections among patients with rheumatoid arthritis (RA) after a third vaccine dose [10]. Furthermore, Parameswaran et al. identified factors such as increasing age, male gender, pre-existing medical comorbidities, and daily exposure to COVID-19 patients as associated with the increased severity of COVID-19 infection after vaccination [11]. Understanding these associations is critical for developing effective risk-reduction strategies and targeted care for individuals at higher risk of severe breakthrough illnesses.

Given the important role of healthcare workers in providing uninterrupted medical services during a pandemic, there is a great need to identify predictors of breakthrough infections within this specific group. By identifying such predictors, preventative measures can be optimized to ensure the well-being and safety of healthcare workers, enabling them to continue their essential work without compromising their health or the health of their patients. In the current study, we aimed to identify the predictors of breakthrough infections in healthcare workers.

## 2. Materials and Methods

### 2.1. Study Cohort

This study is a subanalysis of the data collected during the TüSeRe:exact study (Tübinger Monitoring Studie zur exakten Analyse der Immunantwort nach Vakzinierung) [12]. The TüSeRe:exact study aimed to gather information on longitudinal changes in antibody levels after COVID-19 vaccinations in healthcare workers. The study was registered in the German Clinical Trials Register under the registration number DRKS00029013 (https://drks.de/search/de/trial/DRKS00029013, accessed on 27 December 2023). Employees from the University Hospital Tübingen (n = 10,686), the Center for Clinical Transfusion Medicine (n = 105), and the NMI Natural and Medical Sciences Institute Reutlingen (n = 172) were invited via email and other communication channels to participate in the study [12]. Participants were required to schedule appointments through an online study platform, where they could also complete surveys and access antibody measurements.

Out of 10,963 employees, 1140 participated in the TüSeRe:exact study. However, 94 participants were excluded due to missing data. The participation rate was 10.4%. From the total participants in the TüSeRe:exact study (n = 1046), we included, for the current analysis, study participants who had received three vaccinations and had not been infected before the third vaccination. These participants were invited to complete an online questionnaire detailing their age, gender, time of vaccination, type of vaccines received, comorbidities, medications, and any local or systemic side effects experienced after vaccination. Participants were also invited by email to complete a further online questionnaire on the study website, where they could provide information regarding any breakthrough infections they may have experienced. They were also asked about breakthrough infections at each of their blood sampling visits. Breakthrough infections were defined as laboratory (PCR or rapid antigen test)-confirmed SARS-CoV-2 infections that occurred after being fully vaccinated for at least 14 days [6].

### 2.2. Statistical Analyses

Statistical analysis was conducted using SPSS Version 29 (IBM Inc., Armonk, NY, USA) and GraphPad Prism, Version 8.0 (GraphPad, La Jolla, CA, USA). The Kolmogorov–Smirnov test was used to assess the normality of the data. If the data were normally distributed a t-test would be used to analyze the results. If the data did not follow a normal distribution, the Mann–Whitney test was used. Normally distributed data were presented as the mean ± standard deviation (SD), and non-normally distributed data were presented as the median with an interquartile range (IQR). Categorical parameters were compared with Fischer’s exact test. Univariate Cox regression analysis with adjustment for age and sex was used to investigate the association between participant characteristics and breakthrough infections. Hazard ratios (HRs) with 95% confidence intervals (95%CIs) were calculated. A *p* < 0.05 was considered statistically significant.

## 3. Results

The study cohort consisted of 629 (497 [79%] female and 132 male [21%]) healthcare workers with a mean age of 44.9 ± 12.4 years. A total of 241 (38%) participants experienced a breakthrough infection during the follow-up. The frequency of breakthrough infections was 39.2% (195/497) among female participants and 34.8% (46/132) among male participants (*p* = 0.357). The median time for a breakthrough infection was 114 days (IQR 88–162 days) post the third vaccine dose. Infection was confirmed in 163 (67%) participants via PCR and in 25 (10%) participants using a rapid antigen test. The type of test was not provided in 53 (23%) participants. None of the participants required hospitalization for breakthrough infection.

The mean age of the participants with and without a breakthrough infection was not statistically different (43.7 ± 12.2 years vs. 45.7 ± 12.5 years; *p* = 0.580) (Table 1). However, the percentage of participants under the age of 45 was higher in those with a breakthrough infection (55.2% vs. 46.1%, *p* = 0.027). Gender distribution was similar between both groups. Among comorbidities, the percentage of participants with cardiovascular disease was significantly lower among participants with a breakthrough infection (15.5% vs. 8.7%; *p* = 0.014). The frequency of other comorbidities reported by study participants was not different between the groups (Table 1). Compared to those with a breakthrough infection, the percentage of participants using antihypertensives was significantly lower among participants with a breakthrough infection (14.4% vs. 7.1%; *p* = 0.005). Both groups were similar in terms of other medications reported by participants (Table 1). Finally, the intake of painkillers after the first vaccination was higher in participants with a breakthrough infection than in participants without a breakthrough infection (34% vs. 26.5%; *p* = 0.045; Table 1).

**Figure 1:**
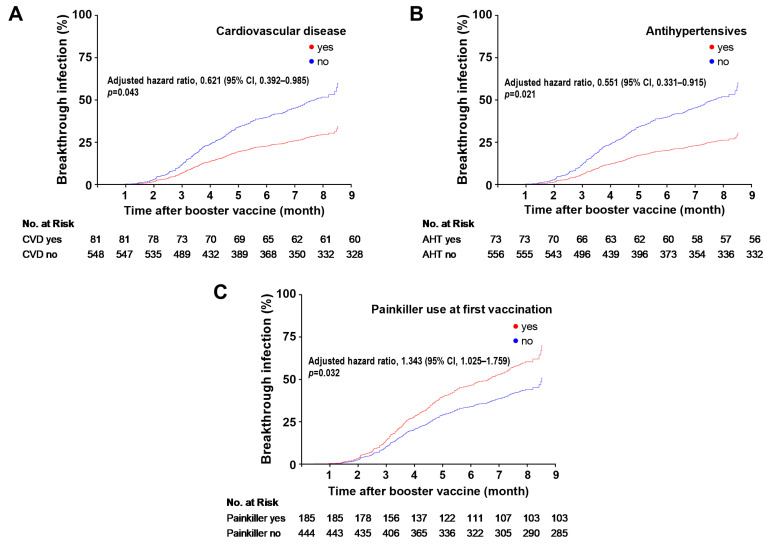
The cumulative incidence of breakthrough infections was examined in relation to cardiovascular disease (Panel **A**), the use of antihypertensive medication (Panel **B**) and painkillers around the time of the first vaccination (Panel **C**). Hazard ratios were calculated using Cox regression analyses with adjustments for age and sex. Breakthrough infections were defined as laboratory-confirmed SARS-CoV-2 infections occurring at least 14 days after the booster vaccination. Abbreviations: CVD= cardiovascular disease; AHT = antihypertensive medication; CI = confidence interval.

To examine the association between participant characteristics and the time to breakthrough infection, a Cox regression proportional hazards model was used (Table 2). After adjustment for age and sex, participants with cardiovascular disease had a lower hazard ratio for breakthrough infections than participants without cardiovascular disease (hazard ratio (95%CI) = 0.621 (0.392–0.985); *p* = 0.043; Figure 1). We found no association between breakthrough infections and other comorbidities (Table 2). Among medications, the use of antihypertensive agents was associated with a lower hazard ratio for breakthrough infections (hazard ratio (95%CI) = 0.551 (0.331–0.915); *p* = 0.021; Figure 1). In contrast, the other medications studied were not associated with breakthrough infections (Table 2). Data on the type of antihypertensive drugs were available in 55 out of 73 participants (75%) taking antihypertensive drugs (Table 3). Although the remaining 18 participants reported taking antihypertensive medication, they did not specify the type. Most of the study participants who did report their medication were using either an angiotensin II receptor inhibitor (n = 25; 34%) or ACE2 inhibitor (n = 18; 25%).

We identified no association between the type of vaccine and breakthrough infections in the first, second, or third vaccine dose (Table 2). By contrast, the use of analgesics after the first (hazard ratio (95%CI) = 1.343 (1.025–1.759); *p* = 0.032; Figure 1), but not after the second or third dose, was associated with an increased risk of breakthrough infections (Table 2).

## 4. Discussion

In this study, we investigated the association between various characteristics and breakthrough infections among healthcare workers. Age was a significant factor in the analysis of breakthrough infections, with participants aged 45 and older exhibiting a notably lower incidence of breakthrough infections in comparison to those under 45 years old. Similarly, Bedston et al. reported an inverse relationship between advancing age and susceptibility to breakthrough infection after both the second and third vaccine doses [13]. This phenomenon could potentially be attributed to varying levels of adherence and protective measures among different age groups, with adherence increasing with age [14], leading to potentially reduced breakthrough infections among older healthcare workers. Differences in the degree of social interactions between different age groups in the healthcare sector could be a possible explanation for this. As the likelihood of encountering a breakthrough infection is closely linked to the possible origins of exposure, increased social interactions may have contributed to higher breakthrough infections. A similar phenomenon was seen among households with children, where the increased potential to carry and spread the virus resulted in a higher incidence of breakthrough infections [13].

A number of studies investigated the association between comorbidities and the risk of breakthrough infections [9,15]. The risk of the SARS-CoV-2 breakthrough infection and the odds of subsequent hospitalization were greater among vaccinated patients with diabetes, chronic lung disease, chronic kidney disease (CKD), and immunocompromising conditions [9,15]. Interestingly, our data found an association between cardiovascular disease and a lower risk of breakthrough infections. In the Cox regression hazard model, we adjusted our data with age and gender. This finding raises intriguing questions and merits further research. One explanation for a reduced breakthrough infection rate in healthcare workers with cardiovascular disease or under antihypertensive therapy might be that individuals in these groups are more vigilant in adhering to protective measures. This heightened awareness of the increased risk of severe infection could lead to a more thorough implementation of preventive strategies, such as consistent mask-wearing, frequent hand hygiene, and strict adherence to physical distancing guidelines, thereby reducing the likelihood of exposure to the virus and subsequent breakthrough infections.

Soegiarto et al., on the other hand, found in a retrospective study that hypertension is associated with a higher risk of breakthrough infections in healthcare workers receiving two doses of an inactivated viral vaccine against SARS-CoV-2 [16]. On the other hand, they did not find any association between cardiovascular disease and breakthrough infection. The differences in the populations studied, including age and gender distribution, as well as variations in the type and number of vaccinations administered and the timing of the pandemic, may account for the discrepancies between the findings of our study and that of Soegiarto et al. They investigated a relatively younger population (36.4 ± 9.86 vs. 44.9 ± 12.4) with an almost equal gender distribution (46.5% female vs. 79% female). Although they found no difference between age and breakthrough infections, our study found that younger individuals had an increased risk of breakthrough infections. This difference in findings may be explained by the fact that individuals with cardiovascular disease in our study were relatively older than those without. To address this potential confounding factor, we used Cox regression analysis after adjusting for age and sex, and the difference in breakthrough infection rates remained significant in our study. While participants in the study by Soegiarto et al. received two doses of an inactivated vaccine, in our study, participants received three vaccinations of either mRNA or a vector vaccine. The differences in the vaccine used, including its efficacy and duration of protection, might also contribute to the observed variations in breakthrough infection rates and discrepant findings between the two studies. Additional research is warranted to better understand the factors contributing to these differences.

The impact of analgesic and antipyretic agents on the immune responses prompted by COVID-19 vaccination remains a relatively unexplored field [17]. Most commonly used COVID-19 vaccines have high reactogenicity [12], resulting in reports of frequent usage in antipyretics/analgesics post-vaccination. It is speculated that the use of antipyretics/analgesics may impact vaccine efficacy [17]. Notably, the administration of paracetamol or ibuprofen to children for antipyretic prophylaxis subsequent to routine pediatric vaccinations has been linked to diminished immunogenicity [18]. In a phase 1/2 study, the preemptive utilization of acetaminophen within a span of 2 days post-vaccination with ChAdOx nCoV19 (University of Oxford, Oxford, UK)alleviated acute vaccine-related symptoms without compromising the overall immunogenicity [19]. An intriguing finding that emerged from our investigation was the increased likelihood of breakthrough infections associated with analgesic use during the first vaccination: a pattern that was not replicated during the second or third vaccination. This observation raises significant questions and underscores the necessity for further in-depth exploration into this specific topic. However, the association between the use of analgesics after the first dose and an increased risk of breakthrough infections requires cautious interpretation and warrants additional investigation to understand potential mechanisms.

We did not find any significant differences in breakthrough infections related to the type of vaccine administered, indicating overall vaccine efficacy across different vaccine formulations and combinations.

Early in the pandemic, it was suggested that antihypertensive drugs (e.g., ACE2 blockers) might lead to the increased expression of ACE2 upregulation, which could increase susceptibility to infection. In clinical practice, two commonly used antihypertensive drugs are ACE inhibitors (ACEIs) and angiotensin II receptor blockers (ARBs). ACEIs inhibit the production of angiotensin II (Ang II) by blocking ACE, while ARBs work by preventing the binding of Ang II to the AT1R receptor [20]. Interestingly, both ACEIs and ARBs were shown to upregulate ACE2 levels [21]. However, several studies utilizing correlation analyses have indicated that the use of ACEIs/ARBs does not increase the risk or mortality associated with COVID-19 [22,23,24]. Further observational studies have consistently provided similar information, reinforcing the notion that ACEIs/ARBs are not associated with adverse outcomes in COVID-19 patients [25,26]. In particular, the use of ACE inhibitors does not appear to heighten the susceptibility to SARS-CoV-2 infection or the severity of the disease, nor does it increase mortality, as indicated by case-population and cohort studies [27]. Interestingly, in the current study, we identified that breakthrough infections after booster vaccination are lower among healthcare workers on antihypertensive medication. The antihypertensive drugs reported include angiotensin II receptor blockers, ACE2 inhibitors, calcium channel blockers, beta-blockers, and diuretics (Table 3). However, 18 participants (25%) who reported taking antihypertensive medication did not specify the type. Due to missing data, further analysis of the association between breakthrough infections and specific antihypertensive drugs has not been performed. The association between antihypertensive agents and a lower risk of breakthrough infections presents an interesting avenue for exploring the immunomodulatory effects of these medications.

## 5. Study Limitations

Our study has notable limitations. Data collection relied on online surveys, potentially introducing recall and response biases. The survey-based nature of our study inherently introduces the possibility of unmeasured confounders, which are variables that are not accounted for in the statistical analysis but may influence the observed outcomes. As a result, we advise caution in interpreting the findings of our study, recognizing the limitations imposed by the inability to measure and account for all potential confounding variables. While we employed statistical methods to control for known confounders, the presence of unmeasured confounders might still affect the observed associations. Moreover, it is important to emphasize that our findings do not establish a causal relationship between the variables studied but rather indicate an association. Other underlying factors or variables not accounted for in our study could contribute to the observed associations. One other limitation of this study is the low participation rate among invited employees, potentially introducing participation bias into the findings. In this case, individuals who opted not to participate might have different characteristics or behaviors compared to those who did participate. For example, they might have lower health awareness, different attitudes towards health-related surveys, or busier schedules that prevented them from taking part. Finally, the study consisted primarily of healthcare workers, limiting its generalizability to broader demographic groups with diverse SARS-CoV-2 exposure levels.

## 6. Conclusions

This study sheds light on factors influencing the incidence of breakthrough infections in healthcare workers. A lower risk of COVID-19 breakthrough infection was demonstrated in individuals with cardiovascular disease or taking antihypertensive medication. These findings can inform targeted preventive measures and risk management strategies to protect healthcare workers and maintain a resilient healthcare system during the ongoing pandemic. However, the cross-sectional design of this study limits our ability to establish causality between variables, and the potential impact of uncontrolled confounders not included in the analysis cannot be excluded. Therefore, we emphasize the need for further studies with rigorous methodologies to confirm our findings and provide a more comprehensive understanding of the associations identified.

## Figures and Tables

**Table 1:** Characteristics of study participants with and without a breakthrough infection.

			Breakthrough Infection		
Parameter		Total	No	Yes	*p*
N		629	388 (62%)	241 (38%)	-
Age					
	<45 years	312	179 (46.1%)	133 (55.2%)	0.027
	≥45 years	317	209 (53.9%)	108 (44.8%)	
Gender					
	Female	497 (79%)	302 (78%)	195 (81%)	0.357
	Male	132 (21%)	86 (22%)	46 (19%)	
Comorbidities					
Cardiovascular disease		81 (12.9%)	60 (15.5%)	21 (8.7%)	0.014
Neurological disease		12 (1.9%)	8 (2.1%)	4 (1.7%)	0.720
Skin disease		35 (5.6%)	22 (5.7%)	13 (5.4%)	0.883
Blood disease		7 (1.1%)	5 (1.3%)	2 (0.8%)	0.594
Pulmonary disease		21 (3.3%)	14 (3.6%)	7 (2.9%)	0.633
Liver/kidney disease		8 (1.3%)	5 (1.3%)	3 (1.2%)	0.962
Gastrointestinal		18 (2.9%)	13 (3.4%)	5 (2.1%)	0.351
Other chronic disease		113 (18%)	77 (19.8%)	36 (14.9%)	0.119
Tumor		19 (3.0%)	14 (3.6%)	5 (2.1%)	0.275
Medications					
Antihypertensives		73 (11.6%)	56 (14.4%)	17 (7.1%)	0.005
Lipid-lowering agents		11 (1.7%)	7 (1.8%)	4 (1.7%)	0.893
Immunosuppressants		14 (2.2%)	10 (2.6%)	4 (1.7%)	0.448
Anticoagulants		10 (1.6%)	5 (1.3%)	5 (2.1%)	0.444
Antidiabetics		10 (1.6%)	5 (1.3%)	5 (2.1%)	0.444
Pain killers		22 (3.5%)	16 (4.1%)	6 (2.5%)	0.278
Antidepressants		23 (3.7%)	17 (4.4%)	6 (2.5%)	0.219
Thyreostatic agent		107 (7.2%)	64 (16.4%)	43 (17.8%)	0.662
Painkiller use after 1st vaccine		185 (29.4%)	103 (26.5%)	82 (34%)	0.045
Painkiller use after 2nd vaccine		171 (27.2%)	97 (25%)	74 (30.7%)	0.118
Painkiller use after 3rd vaccine		63 (10%)	35 (9%)	28 (11.5%)	0.291
Vaccines					
1st Vaccine					
	AZE	309 (49.1%)	188 (48.5%)	121 (50.2%)	0.225
	BNT	258 (41%)	167 (43%)	91(37.8%)	
	MOD	62 (9.9%)	33 (8.5%)	29 (12%)	
2nd Vaccine					
	AZE	66 (10.5%)	43 (11.1%)	23 (9.5%)	0.639
	BNT	420 (66.8%)	261 (67.3%)	159 (66%)	
	MOD	143 (22.7%)	84 (21.6%)	59 (24.5%)	
3rd Vaccine					
	BNT	384 (61%)	230 (59.3%)	154 (63.9%)	0.248
	MOD	245 (39%)	158 (40.7%)	87 (36.1%)	

**Table 2:** Factors associated with a breakthrough infection. Hazard ratios and 95% confidence intervals were calculated using the Cox proportional hazards model after adjustment for age and sex.

Parameter		Hazard Ratio	95% Confidence Interval		*p*
Comorbidities					
Cardiovascular disease		0.621	0.392	0.985	0.043
Neurological disease		0.944	0.350	2.545	0.909
Skin disease		0.936	0.535	1.638	0.817
Blood disease		0.688	0.171	2.767	0.598
Pulmonary disease		0.871	0.410	1.851	0.720
Liver/kidney disease		1.069	0.342	3.346	0.908
Gastrointestinal		0.736	0.303	1.787	0.498
Other chronic disease		0.772	0.541	1.101	0.153
Tumor		0.694	0.284	1.700	0.424
Medications					
Antihypertensives		0.551	0.331	0.915	0.021
Lipid-lowering agents		1.045	0.381	2.866	0.931
Immunosuppressants		0.719	0.267	1.932	0.513
Anticoagulants		1.461	0.598	3.569	0.406
Antidiabetics		1.585	0.650	3.862	0.311
Pain killers		0.698	0.309	1.575	0.386
Antidepressants		0.623	0.277	1.403	0.254
Thyreostatic agent		1.056	0.755	1.478	0.749
Painkiller use after 1st vaccine		1.343	1.025	1.759	0.032
Painkiller use after 2nd vaccine		1.224	0.927	1.617	0.154
Painkiller use after 3rd vaccine		1.282	0.860	1.911	0.223
Vaccines					
1st Vaccine					
	BNT vs. AZE	0.848	0.643	1.120	0.245
	MOD vs. AZE	1.175	0.780	1.769	0.441
	BNT vs. MOD	0.722	0.475	1.099	0.128
2nd Vaccine					
	BNT vs. AZE	1.016	0.648	1.593	0.946
	MOD vs. AZE	1.113	0.679	1.825	0.670
	BNT vs. MOD	0.912	0.676	1.231	0.549
3rd Vaccine					
	BNT vs. MOD	0.982	0.746	1.293	0.899

**Table 3:** Antihypertensive agents. Data were available for 55 participants, with fifteen participants using two agents and one participant using 3 agents.

Drug Group	n
Angiotensin II receptor inhibitors	25
ACE2 inhibitors	18
Calcium channel blockers	13
Beta-blockers	13
Diuretics	3

## Data Availability

Data may be requested for academic collaboration from the corresponding author.
